# Thiamine Compounds Alleviate Oxidative Stress, Over-Expression of Pro-Inflammatory Markers and Behavioral Abnormalities in a Mouse Predation Model of PTSD

**DOI:** 10.3390/ijms26146627

**Published:** 2025-07-10

**Authors:** Tatyana Strekalova, Anna Gorlova, Joao Costa-Nunes, Aleksandr Litavrin, Johannes P. M. de Munter, Alexei Lyundup, Aleksei Umriukhin, Andrey Proshin, Allan V. Kalueff, Edna Grünblatt, Susanna Walitza

**Affiliations:** 1 Department of Psychiatry and Neuropsychology, Maastricht University, 6211 LK Maastricht, The Netherlands; h.demunter@neuroplast.com; 2 Research and Education Resource Center, Peoples Friendship University of Russia (RUDN University), 117198 Moscow, Russia; gorlova_av@pfur.ru (A.G.); liundup-av@rudn.ru (A.L.); 3 Faculdade de Medicina, Universidade de Lisboa, Campo Grande, 1649-028 Lisboa, Portugal; jpcosta.nunes@gmail.com; 4 Department of Normal Physiology, Sechenov University, 119048 Moscow, Russia; alexlitavrin29@gmail.com (A.L.); alum1@yandex.ru (A.U.); 5 Neuroplast BV, 6222 NK Maastricht, The Netherlands; 6 Laboratory of Cognitive Psychophysiology, Federal Research Center for Innovator and Emerging Biomedical and Pharmaceutical Technologies, 125315 Moscow, Russia; proshin_at@mail.ru; 7 Suzhou Municipal Key Laboratory of Neurobiology and Cell Signaling, Department of Biosciences and Bioinformatics, School of Science, Xi’an Jiaotong-Liverpool University, Suzhou 215123, China; avkalueff@gmail.com; 8 Department of Child and Adolescent Psychiatry and Psychotherapy, University of Zurich, 8032 Zurich, Switzerland; edna.gruenblatt@kjpd.uzh.ch; 9 Neuroscience Center Zurich, University of Zurich and the ETH Zurich, 8057 Zurich, Switzerland; 10 Zurich Center for Integrative Human Physiology, University of Zurich, 8057 Zurich, Switzerland

**Keywords:** posttraumatic stress disorder (PTSD), oxidative stress, thiamine (vitamin B1), benfotiamine, predator stress, anxiety-like behavior, neuroinflammation, mice, animal model

## Abstract

Experiences of life-threatening stimuli can induce post-traumatic stress disorder (PTSD), which is associated with long-lasting behavioral and neurochemical abnormalities. Despite its increased global incidence, the current treatment options for PTSD remain limited, highlighting the need for novel therapeutic strategies. As oxidative stress and neuroinflammation contribute to PTSD, the use of powerful antioxidants such as thiamine (B1 vitamin) compounds may counteract disease development. Young C57BL/6 mice received thiamine or benfotiamine in drinking water (each at a dose of 200 mg/kg/day) for 21 days, and for the last five days, they were subjected to rat exposure. Mice were studied for anxiety-like behavior, exploration, locomotion, grooming, social interactions, pain sensitivity, brain changes in protein carbonyl (PC), total glutathione (TG), and gene expression of distress and inflammation markers. Rat exposure induced anxiety-like behavior, excessive grooming, and alteration in locomotion, along with other abnormalities. Stressed, untreated mice had elevated levels of PC and TG in the prefrontal cortex, hippocampus, amygdala, and striatum and increased expression of *Il-1β*, *Tnf*, *c-Fos*, *Cox-1*, and *Cox-2*. Treatment with thiamine or benfotiamine significantly ameliorated most of these changes in the stressed groups. Thus, thiamine compounds may have therapeutic potential in patients with PTSD, owing to their antioxidant and anti-inflammatory properties.

## 1. Introduction

Posttraumatic stress disorder (PTSD) is a complex psychiatric condition [1]. The DSM-5-TR criteria state that children and adults over six years of age who experience a traumatic event, such as threatened death or serious injury, may develop a specific set of symptoms that last over one month, cause significant distress or impairment, and are not caused by drugs or other conditions [1,2]. Symptoms include negative emotions, intrusive distressing memories, avoidance, and significant arousal and reactivity change [1,2]. According to the World Mental Health Surveys, 3.9% of the general population has PTSD, which is 5.6% among trauma victims [3]. Trauma-focused psychotherapies, such as cognitive processing therapy and eye movement desensitization and reprocessing, have moderate efficacy, high dropout rates, and limited accessibility [4,5]. Antidepressants, such as selective serotonin reuptake inhibitors (SSRI), sertraline, paroxetine, and venlafaxine, have limited efficacy and serious side effects, such as insomnia, dizziness, headaches, and weight fluctuations [6,7]. Given the global insufficiency of mental healthcare, many affected individuals remain untreated [3], and solutions to bridge the treatment gap in resource-constrained environments require attention [8].

Preclinical rodent models are essential for elucidating the neurobiological mechanisms of PTSD and developing potentially effective novel therapeutic strategies. PTSD models seek to replicate key characteristics of the disorder, including exposure to emotional trauma, significant physiological and emotional reactions to traumatic stimuli, and related neurobiological irregularities [9]. Physical PTSD models often use high-intensity, time-constrained artificial stressors, such as electric shock, restraint, forced swimming, and immobilization, which lack etiological validity [10,11]. Social models such as social defeat, observed aggression, and sensory exposure to dominant conspecifics have high etiological validity but may be difficult to standardize and reproduce [12]. Predation-related stimuli, such as predator exposure, predator-induced psychosocial stress, and predator scent stress, can cause lasting innate fear and anxiety without physical harm in models of ‘psychological’ or emotional stress. Therefore, they can simulate ethologically relevant threats that cause PTSD and are highly reproducible [13,14,15,16,17,18].

One of the predator-based rodent models that has been extensively studied in recent years is the rat exposure model in mice [15,16,17,18,19]. Mice are exposed to a rat, a natural predator of a mouse, so that visual and olfactory contact between the animals is ensured in close proximity, along with physical protection. In male C57BL/6 mice, 5-day rat exposure increases anxiety-like behavior, suppresses hippocampal cell proliferation, elevates the activity of the distress marker glycogen synthase kinase-3 beta (GSK-3β) and increases the concentration of the oxidative stress marker protein carbonyl (PC) [15]. In Tph2^+/−^ mice with partial inactivation of tryptophan hydroxylase-2 (Tph2), a key brain enzyme in serotonin synthesis [20], rat exposure also induced extreme aggressive behavior regardless of sex that was accompanied by disturbed dopamine- and noradrenaline-ergic brain systems and expression of stress markers in the brain [16,17,18,19].

Other predation paradigms have been shown to recapitulate key behavioral symptoms of PTSD, as well as associated with elevated markers of oxidative stress and neuroinflammation [21]. For example, male Sprague-Dawley rats exposed to a cat, demonstrated increased total reactive oxygen species (ROS) concentrations in the blood, along with elevated levels of pro-inflammatory cytokines, interleukin-1β (IL-1β), interleukin-6 (IL-6), and tumor necrosis factor (TNF), in both plasma and brain regions such as the hippocampus, prefrontal cortex, and amygdala [22]. In another study, male Sprague-Dawley rats subjected to restraint stress followed by forced swimming exhibited increased hippocampal gene expression and protein concentrations of IL-1β, IL-6, and TNF [22,23]. In this model, a reduction in hippocampal levels of reduced glutathione (GSH), as well as a decreased ratio of reduced glutathione to glutathione disulfide (GSH/GSSG), indicating enhanced oxidative stress levels were found [24].

Clinical data are keeping with these mechanistic observations. A recent meta-analysis evidenced a strong link between PTSD and elevated inflammatory and oxidative stress markers [25]. Affected individuals had elevated blood levels of IL-6 and TNF [26,27,28,29,30], as well as increased content of malondialdehyde (MDA), a marker of oxidative stress, and reduced levels of antioxidant enzymes paraoxonase-1 and catalase [31,32,33]. Pro-inflammatory responses and oxidative stress can activate microglia and astrocytes, impair mitochondrial function, damage DNA, and suppress neurogenesis. These cellular disruptions in brain regions associated with fear processing and memory formation, such as the amygdala and hippocampus, may initiate maladaptive neural circuits, thereby facilitating PTSD onset [34,35,36].

Thus, available findings underscore the growing recognition of oxidative stress as a potential therapeutic target for PTSD. However, despite the solid rationale for developing such therapies, this issue has not yet been adequately addressed [37]. Recent preclinical studies have explored some novel antioxidant therapeutic compounds for PTSD, including those that target potassium channels [38], glucocorticoid receptor pathways [39], and drug candidates targeting brain-derived neurotrophic factor (BDNF) and tropomyosin receptor kinase B (TrkB) signaling [40]. However, these studies are scarce, and the potential of pharmacological antioxidant therapy for PTSD remains largely underexplored. Here, we examined the possible beneficial effects of thiamine compounds, powerful antioxidants [41,42]. Using rat exposure stress in mice, a model of PTSD, we aimed to investigate the behavioral and molecular effects of chronic dosing with thiamine (vitamin B1) and its lipid-soluble derivative with high bioavailability benfotiamine, which demonstrated antioxidant, anti-inflammatory, and neuroprotective properties in in vivo and in vitro models [15,19,43,44,45,46,47,48].

Thiamine and benfotiamine have been shown to mitigate oxidative stress, inhibit reactive oxygen species (ROS), and suppress NF-κB-mediated inflammation by stabilizing mitochondrial function [49,50]. Benfotiamine has been demonstrated to modulate the PI3K–Akt–GSK-3β signaling pathway, enhance mitochondrial biogenesis via the PGC-1α mechanism, and elevate cellular glutathione levels by facilitating the synthesis of GSH, a principal intracellular antioxidant that neutralizes free radicals [46]. These characteristics of thiamine medications are likely responsible for the advantageous effects of vitamin B1 supplementation in various neurological and metabolic disorders, such as Alzheimer’s disease [51] and diabetic neuropathy [52,53].

We previously showed that in a modified swim test model of adverse contextual learning, a two-week thiamine pre-treatment of C57BL/6 mice at a dose of 200 mg/kg/day alleviated depressive-like and anxiety-like behaviors, reduced blood corticosterone levels, decreased brain MDA levels, and normalized elevated brain *Il-1β*, *Tnf*, *Cox-1*, and *Gsk-3β* expression, which was induced by stress [43,45,47]. The administration of thiamine or benfotiamine at the same dose for three weeks to male BALB/c mice counteracted stress-induced aggressive behavior and helplessness, reversed brain upregulation of PC and total glutathione (TG), and ameliorated brain gene expression of AMPA receptor subunits and plasticity markers polysialylated-neural cell adhesion molecule (PSA-NCAM), postsynaptic density protein 95 (PSD95), and β-catenin [44]. Chronic administration of dibenzoyl thiamine (DBT), a highly bioavailable thiamine derivative, at doses of 25 or 200 mg/kg/day to C57BL/6 mice exerted antidepressant-like and antioxidant effects in the ultrasound stress paradigm [41]. Finally, a 6-week treatment with DBT (200 mg/kg/day) exerted neuroprotective effects in a FUS-tg mouse model of amyotrophic lateral sclerosis, lowering plasma levels of IL-1β and GSK-3β, as well as their brain expression [41,48].

In the present study, we aimed to examine behavioral and molecular effects of administration of thiamine and benfotiamine in a rat exposure model of PTSD, in a context of a perspective of clinical application of these compounds whose good tolerability is well documented in numerous clinical studies by now. Here, young C57BL/6 mice received thiamine or benfotiamine in drinking water, each at a dose of 200 mg/kg/day, for 21 days, and for the last five days were subjected the predation. The choice of dose was based on previous studies showing their efficacy in stress models and the absence of side effects, [15,44]. Additionally, the dosage was established based on FDA-recommended guidelines for rodent translational studies, using a 13-fold increase over the recommended human dose to ensure proper scaling for murine physiology.

Mice were studied for anxiety-like behavior, exploration, locomotion, grooming, and social interactions using novel cage and open field tests and a resident–intruder paradigm. Additionally, we investigated pain sensitivity using the tail flick test, as pain processing is known to be altered in patients [54]. These data were related to changes in brain oxidative stress; therefore, the concentrations of PC and TG were evaluated in the prefrontal cortex, hippocampus, amygdala, striatum, and dorsal raphe. TG levels were used as a marker of neuroprotection against oxidative stress [55,56]. The same brain structures were also investigated for the gene expression of *Il-1β*, *Tnf*, *c-Fos*, *Cox-1*, and *Cox-2*, markers of distress and inflammation related to oxidative stress [57,58].

Although thiamine compounds have been previously explored in a pilot study involving a rat exposure model, the present study is the first to assess a broad range of behavioral parameters, including anxiety, aggression, and pain sensitivity. In addition, this study aimed to investigate the potential changes in oxidative stress markers in numerous brain areas that were not explored in applied here PTSD model. In previous studies on thiamine compounds, the potential effect of their administration was limited to the hippocampus and prefrontal cortex. Thus, this study is an inaugural comprehensive investigation into the effects of thiamine compounds using a predator-based mouse model of PTSD, expanding upon prior research by evaluating a wide array of behavioral outcomes, along with oxidative stress and inflammatory markers, in various brain regions, including the amygdala and the striatum.

## 2. Results

### 2.1. Chronic Dosing with Thiamine or Benfotiamine Ameliorated Parameters on Anxiety-like Behavior, Locomotor Activity, and Pain Sensitivity Altered by Rat Exposure

**Figure 1:**
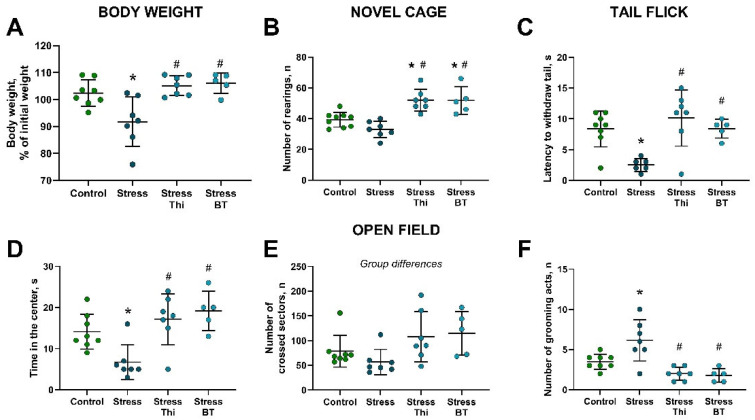
Rat exposure stress altered body weight and anxiety-like and locomotor parameters, which were ameliorated by thiamine or benfotiamine treatment. (**A**) Body weight, calculated as a percentage of the initial weight. (**B**) Number of rearings in the novel cage test. (**C**) Latency to tail withdrawal in the tail flick test. (**D**) Time spent in the center of the open field. (**E**) Total number of crossed sectors in the open field. (**F**) Total number of grooming acts in the open field. * *p* < 0.05 vs. control group, # *p* < 0.05 vs. stressed non-treated group, two-way ANOVA and post hoc Tukey’s test, Kruskal–Wallis test. Bars are Mean ± SD. Thi—thiamine, BT—benfotiamine. Control group, n = 8, stressed non-treated group, n = 7, stressed thiamine-treated group, n = 7, stressed benfotiamine-treated group, n = 5.

Body weight, expressed as a percentage of the pre-stress baseline values, differed significantly between the groups (F = 8.215, *p* = 0.0007, two-way ANOVA). This parameter was significantly decreased in the non-treated stressed group compared to the control group (*p* = 0.101, Tukey’s test) and in the stressed groups that received thiamine or benfotiamine (*p* = 0.0016 and *p* = 0.0022, respectively; Figure 1A). The number of rearings in the novel cage was significantly different between groups (F = 14.65, *p* < 0.0001, one-way ANOVA). This measure was significantly higher in the groups treated with thiamine and benfotiamine than in both the control group (*p* = 0.0031 and *p* = 0.0089, respectively) and non-treated stressed mice (*p* < 0.0001 and *p* = 0.0002, respectively, Tukey’s test; Figure 1B). After removing one outlier from the stressed non-treated group following the recommendations of the Robust Regression and Outlier Removal methods, significant group differences were observed in the latency to tail withdrawal in the tail-flick test (F = 7.688, *p* = 0.0011, one-way ANOVA). This parameter was significantly lower in the stressed non-treated group than in the non-stressed group (*p* = 0.0078, post hoc Tukey’s test) and in stressed mice treated with thiamine (*p* = 0.0008) or benfotiamine (*p* = 0.0184; Figure 1C).

The time spent in the center of the open field was significantly different between groups (F = 7.97, *p* = 0.0008, one-way ANOVA). This parameter was significantly lower in the untreated stressed group than in the control group (*p* = 0.0362, Tukey’s test). Treatment with thiamine or benfotiamine significantly increased this measure relative to the untreated stressed group (*p* = 0.0031 and *p* = 0.0013, respectively; Figure 1D). ANOVA indicated significant group differences in the number of crossed sectors (F = 3.104, *p* = 0.0464; Figure 1E); however, post hoc Tukey’s test did not identify any significant changes (*p* > 0.05).

A significant difference between the groups was revealed in the number of grooming acts (F = 11.74, *p* < 0.0001, one-way ANOVA). This indicator was significantly elevated in the non-treated stressed group compared to the controls (*p* = 0.012, Tukey’s test) and was significantly ameliorated by both thiamine (*p* = 0.0002) and benfotiamine (*p* = 0.0003; Figure 1F). No significant differences were observed in aggression scores (Supplementary Figure S1).

### 2.2. Increased Brain Concentration of Oxidative Stress Markers in Mice Subjected to Rat Exposure Stress and Normalizing Effects of Thiamine Compounds

**Figure 2:**
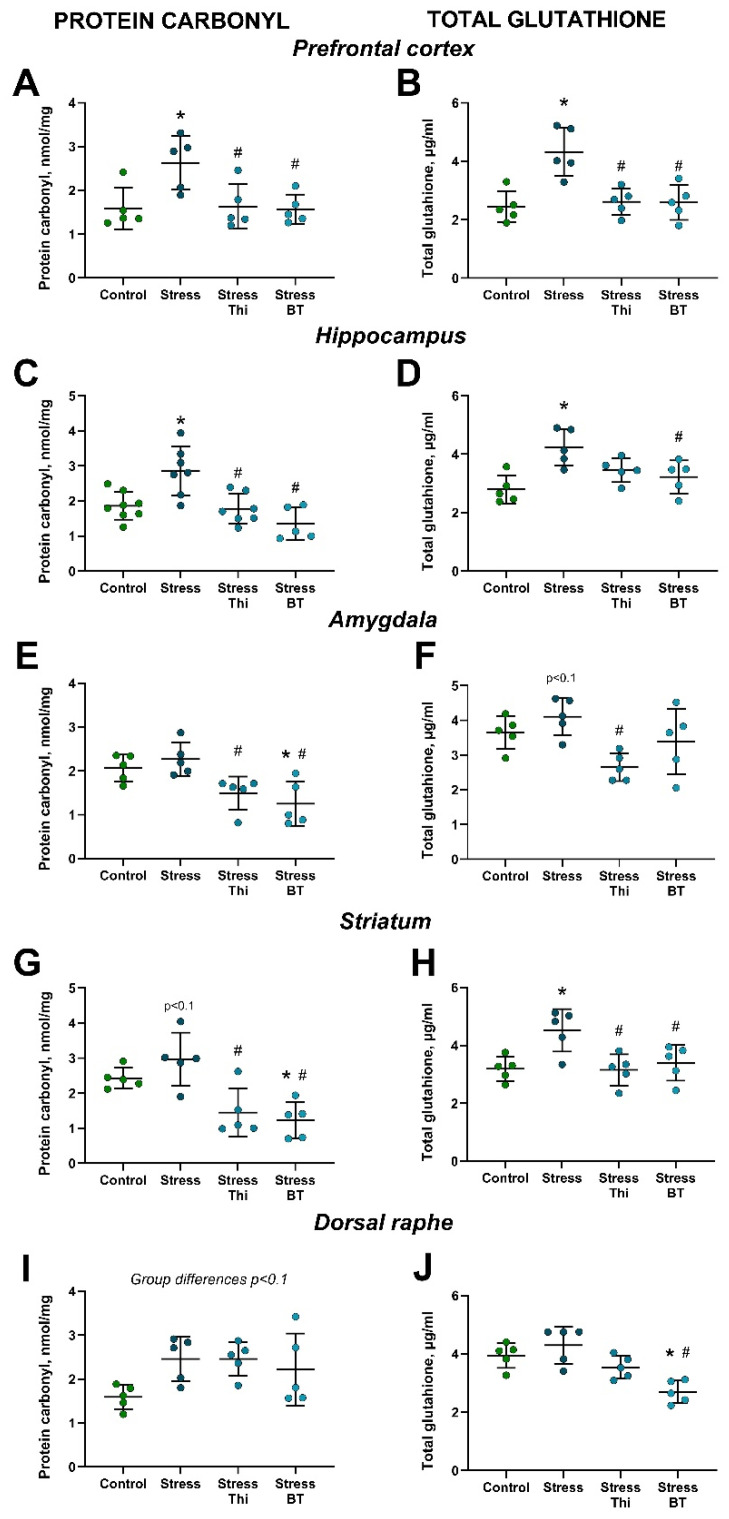
Increased brain oxidative stress markers in mice subjected to rat exposure stress and the effects of thiamine compounds. (**A**) PC content in the prefrontal cortex. (**B**) TG concentration in the prefrontal cortex. (**C**) PC content in the hippocampus. (**D**) TG content in the hippocampus. (**E**) PC content in the amygdala. (**F**) TG concentration in the amygdala. (**G**) PC concentration in the striatum. (**H**) TG content in the striatum. (**I**) PC content in the dorsal raphe. (**J**) TG levels in the dorsal raphe. * *p* < 0.05 vs. control group, # *p* < 0.05 vs. stressed non-treated group, two-way ANOVA and post hoc Tukey’s test. Bars are Mean ± SD. PC—protein carbonyl, TG—total glutathione, Thi—thiamine, BT—benfotiamine. All n = 5.

ANOVA revealed significant group differences in PC concentrations in the prefrontal cortex (F = 5.468, *p* = 0.0088, one-way ANOVA). This measure was significantly increased in the non-treated stressed group compared to the controls (*p* = 0.0197, Tukey’s test) and ameliorated by both thiamine and benfotiamine (*p* = 0.0264 and *p* = 0.0178, respectively; Figure 2A). The PC levels in the hippocampus differed significantly between the groups (F = 9.883, *p* = 0.0002). Similarly, this indicator was significantly increased in the non-treated stressed group compared to the controls (*p* = 0.0051, Tukey’s test) and the stressed group treated with both thiamine and benfotiamine (*p* = 0.0032 and *p* = 0.0002, respectively; Figure 2C). Significant group differences were observed for PC content in the amygdala (F = 7.04, *p* = 0.0031, one-way ANOVA). It was significantly decreased in the stressed benfotiamine-treated group compared to both the control and stressed non-treated groups (*p* = 0.0259 and *p* = 0.0051, respectively; Tukey’s test). It was also significantly lower in the stressed thiamine-treated group than in the stressed untreated mice (*p* = 0.0339; Figure 2E). Group differences were revealed by ANOVA for the PC concentration in the striatum (F = 9.467, *p* = 0.0008). A strong trend for its increase in the stressed non-treated group compared to that in the control group was found (*p* = 0.0798, Tukey’s test). The stressed thiamine-treated group demonstrated a significant decrease in PC content in the striatum compared to the non-treated stressed mice (*p* = 0.0047). Stressed benfotiamine-treated mice had a significantly lower level than that of both the control and non-treated stressed groups (*p* = 0.027 and *p* = 0.015, respectively; Figure 2G). A strong trend for group differences in PC levels in the dorsal raphe nucleus was observed (F = 2.904, *p* = 0.067; Figure 2I).

TG levels in the prefrontal cortex differed significantly between groups (F = 10.32, *p* = 0.0005). It was significantly elevated in the non-treated stressed group compared to the control group (*p* = 0.001, Tukey’s test) and the stressed groups treated with thiamine or benfotiamine (*p* = 0.0025 and *p* = 0.0022, respectively; Figure 2B). In the hippocampus, ANOVA revealed significant group differences in TG levels (F = 6.606, *p* = 0.0041). Specifically, it was significantly higher in the untreated stressed group than that in the control group (*p* = 0.0026, Tukey’s test). This parameter was significantly lower in the stressed group treated with benfotiamine (*p* = 0.0347; Figure 2D), but not thiamine (*p* = 0.125), compared to the stressed non-treated group. TG content differed significantly between the groups in the amygdala (F = 4.732, *p* = 0.0151, one-way ANOVA). A strong trend for its increase in the stressed non-treated group compared to that in the control group was observed (*p* = 0.0961, Tukey’s test). This measure was significantly lower in thiamine-treated (*p* = 0.0099; Figure 2F) but not benfotiamine-treated mice (*p* = 0.2986) than in stressed non-treated groups. Significant group differences were revealed by ANOVA for the TG concentration in the striatum (F = 6.037, *p* = 0.006). Specifically, it was significantly elevated in the non-treated stressed group compared to that in the control group (*p* = 0.012, Tukey’s test), stressed thiamine-treated group (*p* = 0.01), and stressed benfotiamine-treated group (*p* = 0.036; Figure 2H). Significant group differences were found in TG content in the dorsal raphe (F = 10.59, *p* = 0.0004, one-way ANOVA). It was significantly lower in the stressed benfotiamine-treated group than in both the control and stressed untreated groups (*p* = 0.0036 and *p* = 0.0003, respectively; Figure 2J).

### 2.3. Upregulated Brain Gene Expression of Inflammatory Markers, Cyclooxygenases, and c-Fos in Mice Subjected to Rat Exposure Stress Is Counteracted by the Treatment with Thiamine Compounds

**Figure 3:**
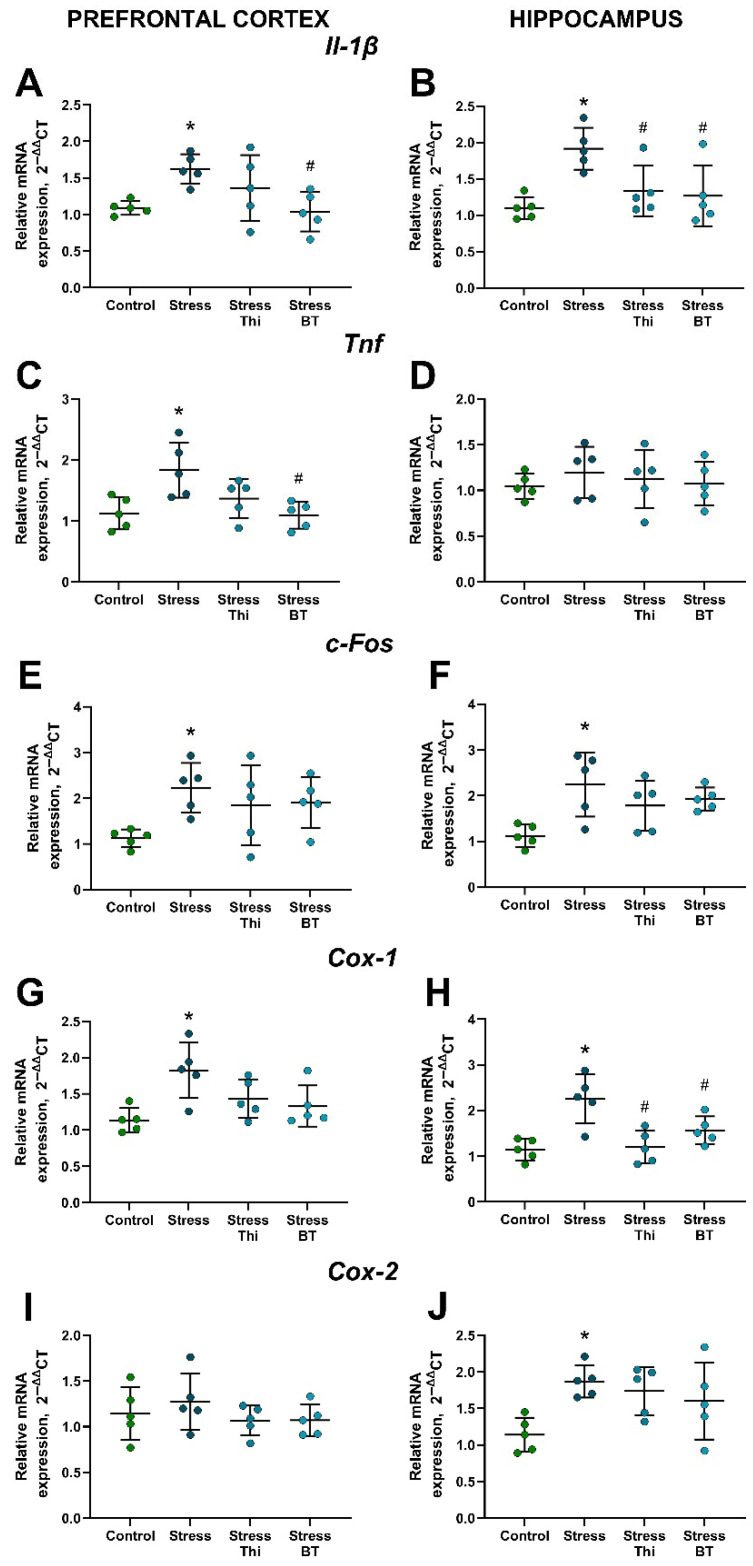
Increased brain gene expression of inflammatory markers, cyclooxygenases, and c-Fos in mice subjected to rat exposure stress and the effects of thiamine compounds. (**A**) *Il-1β* expression in the prefrontal cortex. (**B**) *Il-1β* expression in the hippocampus. (**C**) *Tnf* expression in the prefrontal cortex. (**D**) *Tnf* expression in the hippocampus. (**E**) *c-Fos* expression in the prefrontal cortex. (**F**) *c-Fos* expression in the hippocampus. (**G**) *Cox-1* expression in the prefrontal cortex. (**H**) *Cox-1* expression in the hippocampus. (**I**) *Cox-2* expression in the prefrontal cortex. (**J**) *Cox-2* expression in the hippocampus. * *p* < 0.05 vs. control group, # *p* < 0.05 vs. stressed non-treated group, two-way ANOVA and post hoc Tukey’s test. Bars are Mean ± SD. Thi—thiamine, BT—benfotiamine. All n = 5.

Significant group differences were revealed for *Il-1β* expression in the hippocampus and prefrontal cortex (F = 6.310, *p* = 0.005 and F = 4.456, *p* = 0.0186, respectively, one-way ANOVA). Specifically, its expression was significantly increased in the hippocampus (*p* = 0.0043, Tukey’s test) and prefrontal cortex (*p* = 0.042) of non-treated stressed mice compared to the control group. Hippocampal expression of *Il-1β* was significantly decreased in both thiamine- and benfotiamine-treated stressed mice compared to that in the non-treated stressed group (*p* = 0.0457 and *p* = 0.0239, respectively; Figure 3B). In the prefrontal cortex, this measure was ameliorated by benfotiamine (*p* = 0.0244), but not by thiamine (*p* = 0.489; Figure 3A). There were no significant group differences in *Tnf* expression in the hippocampus (F = 0.337, *p* = 0.798, one-way ANOVA; Figure 3D). At the same time, *Tnf* expression was observed in the prefrontal cortex (F = 5.553, *p* = 0.0083). This parameter was significantly higher in the non-treated stressed group than in the control group (*p* = 0.0154, Tukey’s test) and significantly lower in the group treated with benfotiamine (*p* = 0.0114), but not thiamine (*p* = 0.1446; Figure 3C), compared to non-treated stressed mice.

The ANOVA revealed significant group differences in *c-Fos* expression in the hippocampus and prefrontal cortex (F = 4.853, *p* = 0.0138 and F = 3.08, *p* = 0.0574, respectively). Specifically, *c-Fos* expression was increased in both the hippocampus (*p* = 0.0094, Tukey’s test; Figure 3E) and prefrontal cortex (*p* = 0.403; Figure 3F) in the stressed non-treated group compared to the controls. Stressed thiamine- and benfotiamine-treated mice did not differ significantly from the controls in these measures (all *p* > 0.05, Tukey’s test).

Significant group differences were revealed in *Cox-1* expression in the hippocampus (F = 9.419, *p* = 0.0008, one-way ANOVA). This indicator was elevated in the non-treated stressed group compared to the controls (*p* = 0.0012, Tukey’s test) and ameliorated by thiamine and benfotiamine (*p* = 0.002 and *p* = 0.0451, respectively; Figure 3H). Significant group differences were also found in *Cox-1* expression in the prefrontal cortex (F = 5.149, *p* = 0.0111), which was increased in the stressed untreated group compared to that in control mice (*p* = 0.0055; Figure 3G). Thiamine and benfotiamine did not significantly lower this measure (*p* = 0.175 and *p* = 0.0641, respectively), suggesting that there was a strong trend for its decrease after benfotiamine treatment. *Cox-2* expression in the hippocampus differed significantly between the groups (F = 4.138, *p* = 0.0238, one-way ANOVA) and was significantly higher in the non-treated stressed group than in the control group (*p* = 0.0208, Tukey’s test; Figure 3I). No significant group differences were found in *Cox-2* expression in the prefrontal cortex (F = 0.796, *p* = 0.514; Figure 3J).

## 3. Discussion

The current study demonstrates that the administration of both thiamine and its analog benfotiamine exerts comparable ameliorative behavioral, biochemical, and molecular effects, counteracting the effects of predation in a mouse model of PTSD. Consistent with previous findings, non-treated mice subjected to rat exposure to predation stress displayed significant loss of body weight, increased parameters of anxiety-like behavior, suppressed locomotor activity, and novelty exploration. In addition, our study revealed strikingly increased grooming, an indicator of stress-related behavioral invigoration and emotional reactivity in rodents [59,60]. These behavioral abnormalities were accompanied by significant increases in markers of oxidative stress across all investigated brain structures, including the prefrontal cortex, hippocampus, amygdala, and striatum, and an upregulation of the stress marker *c-Fos*, and markers of inflammation of *Il-1β*, *Tnf*, *Cox-1*, and *Cox-2* in the brain. Most behavioral and molecular changes were abolished by pretreatment with thiamine or benfotiamine, including weight loss, an indicator of stress [58], and a state of systemic inflammation [60,61].

Here, we reported behavioral changes in mice, such as anxiety and abnormally increased grooming behavior which can be interpreted as parallels of emotional dysregulation, hyperarousal, and hypervigilance in PTSD patients [62,63,64]. We found significant increases in the concentrations of PG and TG in the prefrontal cortex, hippocampus, amygdala, and striatum in the non-treated stressed group, indicating a robust and widespread oxidative stress response in these animals. As discussed above, an increase in oxidative stress markers is an important element in PTSD pathology [25,34]. These changes were similarly prevented in both thiamine- and benfotiamine-treated animals. The upregulation of stress marker *c-Fos* and markers of inflammation *Il-1β*, *Tnf*, *Cox-1*, and *Cox-2* in the hippocampus and prefrontal cortex shown in the non-treated stressed group is known to be interconnected with elevated oxidative stress [65] and, as discussed, is established as another important feature of PTSD [66,67]. Again, these predation-induced gene expression changes were not observed in the stressed thiamine- or benfotiamine-treated groups.

The evidence of activated neuroinflammatory pathways in the employed mouse PTSD model is consistent with previous observations in stress models. For example, COX-2 is associated with trauma-induced inflammatory responses [68] and stress-induced behavioral abnormalities, including anhedonia [69,70], and treatment with the selective COX-2 inhibitor celecoxib reduces stress-induced changes in behavior, inflammation, and cell death [68,70]. Enhanced *Cox-1* expression was observed in the prefrontal cortex of male C57BL/6 mice subjected to a 10-day stress protocol, which included nighttime rat exposure [71].

This immune response was paralleled by increased *c-Fos* expression in the prefrontal cortex and hippocampus of untreated stressed mice, similar to the results of other rodent stress studies [72,73,74]. The upregulation of *c-Fos*, an immediate early gene and marker of neuronal activation, is a well-established marker of PTSD [74,75,76]. Pharmacological inhibition of IL-1β receptor antagonists or nonsteroidal anti-inflammatory drugs reduces behavioral and inflammatory responses in predator stress in male Sprague-Dawley rats [77,78]. Elevated *c-Fos* expression has been found in the amygdala and hypothalamus in a PTSD rat stress model in mice [75,76], along with increased expression of *Gsk-3β*; the overexpression of these molecules was shown to promote neuroinflammation [79]. Wild-type and Tph2^+/−^ mice exposed to a C57BL/6 background were found to display *Gsk-3β* and *c-Fos* upregulation in the prefrontal cortex and amygdala [16]. Additionally, serum TNF concentrations were elevated in Sprague-Dawley rats exposed to restraint and forced swim stress, whereas treatment with anti-TNF antibodies ameliorated anxiety-like behavior [80].

Treatment with either thiamine compound prevented most of the predation-induced increases in oxidative stress and inflammation markers in the brain. Interestingly, while previous studies revealed superior anti-stress efficacy of benfotiamine in comparison to thiamine [45,46], the current work showed similar effects of the two compounds. However, benfotiamine-treated stressed mice had a significant reduction in *Tnf* and Il-1β expression in the prefrontal cortex, while in the stressed thiamine-treated group, the changes did not reach the level of significance.

This aligns with previous data suggesting that benfotiamine may exert stronger neuroprotective effects than thiamine, largely because of its enhanced lipid solubility and bioavailability [42]. For example, a study in healthy male participants demonstrated that benfotiamine led to higher plasma and cellular levels of TPP despite a lower administered dose and more effectively stimulated transketolase activity, indicating superior cellular efficacy [81]. Beyond its classical role as a thiamine precursor, benfotiamine exhibits pleiotropic neuroprotective effects, including enhancement of mitochondrial function and biogenesis via the PGC-1α pathway [82], modulation of the PI3K/Akt/GSK-3β signaling axis [83], and elevation of antioxidant defenses such as GSH and superoxide dismutase activity [46]. Furthermore, benfotiamine has been shown to suppress NF-κB-mediated inflammation and stabilize mitochondrial integrity [50], supporting its potential as a therapeutic agent in neurodegenerative and stress-related disorders by maintaining redox homeostasis and protecting against oxidative stress.

At the same time, both compounds normalized the PC and TG levels in our study, which is in line with previous reports [25,34]. These findings also corroborate previous studies in which thiamine compounds modulated oxidative damage and inflammatory cytokines in models of chronic stress and genetically determined neurodegeneration [44,47,48].

Given that patients with PTSD display altered pain sensitivity, in which the role of neuroinflammation and oxidative stress is established [84,85], we hypothesized that pain sensitivity might be altered in mice exposed to predation. The present study demonstrated significantly reduced latency to tail withdrawal in the tail-flick test, indicating elevated pain sensitivity after predator exposure in pharmacologically naïve animals. This suggests that rat exposure elevates nociception, consistent with prior research linking enhanced pain sensitivity to PTSD [86,87]. Similar findings have been reported in a rat model of PTSD characterized by foot shock coupled with social isolation, using the von Frey test alongside assessments of thermal and mechanical nociception [88]. Notably, stressed mice treated with either thiamine or benfotiamine exhibited significantly diminished pain sensitivity in the tail-flick test, suggesting the potential normalizing effect of these compounds on stress-induced hyperalgesia.

Here, we did not observe significant group differences in the parameters of aggressive behavior, despite their potential relevance in trauma-related PTSD conditions [89]; however, a trend toward an increased number and duration of attacks was observed in stressed mice treated with thiamine compounds. In the novel cage test, stressed mice that received either compound showed a significantly increased number of rearings, a measure of novelty exploration that is also correlated with stress resilience [90]. Notably, there was a trend in the number of crossed sectors in the open field test in the stressed treated groups, which might be keeping with other findings suggesting anti-stress and anti-anxiety effects of thiamine and benfotiamine in our study. Indeed, prolonged dosing with a low dose of thiamine was previously shown to increase locomotor activity and suppress freezing behavior in the open field in rats subjected to chronic immobilization stress [91]. Similarly, the administration of thiamine tetrahydrofurfuryl disulfide, a lipophilic derivative of thiamine, promoted voluntary wheel-running activity in rats [92]. At the same time, dietary thiamine deficiency resulted in decreased exploratory activity and reduced novelty seeking in male C57BL/6 mice [93]. Thus, data reported here data are consistent with previous findings showing that thiamine compounds can exert anti-stress and anti-anxiety effects that manifest as improved novelty exploration, ambulations, and overall activity in experimental rodents.

Previously reported mitochondrial, antioxidant, and anti-inflammatory mechanisms suggest the therapeutic potential of thiamine and its derivatives in stress and neurodegenerative disorders. Thiamine, particularly in the form of thiamine pyrophosphate (TPP), plays a central role in mitochondrial energy metabolism and cellular redox regulation. As a cofactor for key mitochondrial enzyme complexes such as pyruvate dehydrogenase or α-ketoglutarate dehydrogenase, TPP supports ATP production via the tricarboxylic acid cycle [94]. Additionally, thiamine is essential for the activity of transketolase, a cytosolic enzyme that catalyzes the steps in the pentose phosphate pathway and is crucial for generating NADPH, a cofactor required for maintaining GSH levels and redox homeostasis [94]. In addition, thiamine possesses exerts antioxidant properties, likely mediated by proton donation from its pyrimidine and thiazole rings, enabling it to scavenge ROS and prevent lipid peroxidation [94,95]. Moreover, thiamine contributes to immune modulation by enhancing macrophage phagocytic activity and reducing the secretion of pro-inflammatory cytokines. It also interacts with the tumor suppressor protein p53, influences apoptosis and cell cycle control, and inhibits the formation of advanced glycation end-products (AGEs) associated with cellular dysfunction and tissue damage [96].

Our study suggests that the potential therapeutic efficacy of thiamine and benfotiamine in PTSD patients, given the well-documented clinical evidence of their good tolerability, might soon be implemented in practice. Indeed, thiamine at a dose of 250 mg/day has already been used to treat patients with generalized anxiety disorders [97]. In a triple-blinded randomized placebo-controlled clinical trial, the administration of 300 mg/day of thiamine for four weeks in women with polycystic ovary syndrome reduced anxiety, depression, and somatic symptoms [98]. A recent retrospective cohort study involving 2280 patients showed that an optimal dose of thiamine above 200 mg/day exerts beneficial effects on brain function in patients with traumatic brain injury [99]. Benfotiamine, used at a dose of 600 mg/day in a one-year long randomized, placebo-controlled trial in patients with Alzheimer’s disease, showed good tolerability and therapeutic efficacy that also reduced blood levels of markers of glycation and glycation end-products [51]. A similar treatment applied in patients with type 2 diabetes in a randomized, placebo-controlled study confirmed the excellent tolerability of benfotiamine and significantly ameliorated the progression and symptoms of distal symmetric polyneuropathy. This was accompanied by the suppression of systemic expression of the inflammation marker cluster of differentiation 31 (CD31) and oxidative stress marker superoxide dismutase 2 (SOD2) [52]. A recent study by Jia et al. showed good tolerability of supplementary thiamine and its positive effects on executive function, global cognition, energy metabolism, neurotransmitter synthesis, and protection against oxidative stress in elderly patients [100].

Taken together, the current and previously reported findings suggest the therapeutic potential of thiamine and benfotiamine for the treatment and prevention of PTSD symptoms, which can be a new valuable strategy for pharmacotherapy of this disorder, particularly considering the limitations of currently available therapies for PTSD [6]. Because of the good tolerability of these drugs, our results provide a strong rationale for further clinical investigation of thiamine derivatives as adjunctive or standalone options in patients with PTSD.

## 4. Materials and Methods

### 4.1. Experimental Animals

The experiment utilized male C57BL/6 mice, aged three months, procured from the certified supplier Charles River (Janvier, Evreux, France). Additionally, male BALB/c mice of the same age from the same supplier were employed as counterparts in the resident–intruder test, young male Wistar rats were used for predation stress. The mice were single housed, rats were housed in groups of five prior to experimental period; animals were kept under standard laboratory conditions, with unrestricted access to food and water. A reverse light/dark cycle was implemented, with lights on at 20:00 and off at 08:00. The mice were acclimatized for one week prior to further procedures. All behavioral experiments were conducted during the dark phase, with other potential confounding factors controlled as described elsewhere [101]. Observations of the animals were conducted each morning and evening throughout the experimental period. All experiments were performed in accordance with the European Union’s Directive 2010/63/EU, as well as Portuguese Law-Decrees DL129/92 (July 6th), DL197/96 (October 16th), and Ordinance Port. 131/97 (November 7th). Approval was obtained from the Direccao Geral de Veterinaria, Ministerio da Agricultura, do Desenvolvimento Rural a das Pescas, under license number 685412, DG VGZ/VVP (S. 135), 0421/000/000/2013, and the Universidade de Lisboa regarding animal care and welfare (DGV-2009-10-22-00248216) and ethical permission (02.03.2020). The experiments adhered to the ARRIVE guidelines (http://www.nc3rs.org.uk/arrive-guidelines (accessed on 10 May 2022)). Every effort was made to minimize potential discomfort for the animals, and the study did not include humane endpoints.

### 4.2. Study Flow

**Figure 4:**
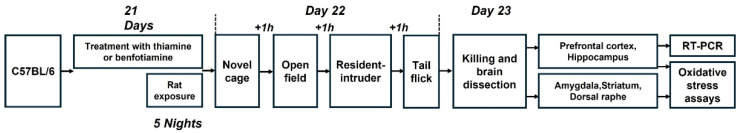
Experimental Timeline and Procedure. Male C57BL/6 mice received thiamine or benfotiamine (200 mg/kg/day via drinking water) for 21 days. During the final five nights, the animals were subjected to rat exposure to stress. Behavioral testing was carried out on day 22 and included the novel cage, open field, resident–intruder, and tail-flick tests. On Day 23, the mice were euthanized for brain dissection. The prefrontal cortex, hippocampus, amygdala, striatum, and dorsal raphe were analyzed for oxidative stress markers. Parts of the prefrontal cortex and hippocampus were utilized for subsequent RNA isolation and quantitative reverse transcription polymerase chain reaction (RT-qPCR) assays.

A total of 27 experimental mice and 19 rats were utilized in this study. The animals were allocated to either a control group (n = 8) or a stressed group. Subgroups of the stressed mice were either left untreated (n = 7) or administered thiamine (n = 7) or benfotiamine (n = 5) at a dosage of 200 mg/kg/day via drinking water, as previously described [15,43,44], for a period of 21 days. The sample size was determined based on previous studies using this PTSD model and/or thiamine compounds, with the aim of balancing statistical power with ethical considerations [15,44]. Group sizes of five to eight have been shown to be sufficient for detecting behavioral and molecular effects, allowing our design to follow the 3Rs principle by limiting animal use without compromising data reliability. Randomization was conducted based on body weight. Stress exposure was applied to the rats during the last five nights, following established protocols [17,19,43,44]. On Day 22, the mice were weighed and subjected to the novel cage, open field, resident–intruder, and tail flick tests, with a minimum inter-trial interval of one hour. On Day 23, all animals were euthanized, and their prefrontal cortex, hippocampus, amygdala, striatum, and dorsal raphe were extracted for fluorometric assays. Portions of the prefrontal cortex and hippocampus were also harvested for reverse transcription quantitative real-time PCR (RT-qPCR) analysis (Figure 4). A sample size of 5 per group was used for both fluorometric and RT-PCR assays, as this number provided sufficient statistical power based on prior studies [15] and allowed all samples to be processed on a single plate, minimizing inter-assay variability. The experimenter remained blind to the group assignments until the data analysis phase.

### 4.3. Rat Exposure Stress

Mice were placed in transparent cylindrical containers (15 cm × Ø 8 cm) and introduced into the rat cage for 15 h exposure sessions conducted from 18:00 to 09:00. These containers, constructed from transparent plastic, featured perforated lids with openings smaller than 0.5 cm in diameter, ensuring physical separation from the rats while permitting visual and olfactory contact [15,16,17,18,19]. Mice had unrestricted access to food and water in their home cages between stress sessions. The timing of the rat exposure model was designed to minimize the effects of food and water deprivation, as the predation period coincided with the light (inactive) phase, during which mice naturally consume virtually no diet [17,101]. Although food deprivation may contribute to overall stress, our previous studies using a similar model demonstrated that C57BL/6 mice do not consume food or water during predation stress, even when available [17,101]. Therefore, while the environment involves multiple stressors, the primary stressor remains the fear of predation.

### 4.4. Novel Cage

In this test, each mouse was placed in a clear plastic cage (14 × 21 × 27 cm) containing a small amount of fresh bedding. For 5 min, the number of rearing events was recorded under red light, as previously described [101,102].

### 4.5. Open Field Test

The open field test was conducted in a square arena (45 × 45 × 45 cm) illuminated with low-intensity white light (5 lux), as previously described [102,103]. Each mouse was placed near a wall, and its behavior was recorded over a 5 min period. Using validated protocols with the EthoVision program (Noldus, Wageningen, The Netherlands), the following parameters were analyzed: number of crossed sectors (5 × 5 cm), time spent in the central area (15 × 15 cm), and number of grooming acts as described elsewhere [17,104].

### 4.6. Resident–Intruder Test

The resident–intruder test was conducted as previously described [17,44]. Age-matched, group-housed naïve BALB/c male mice served as intruders and were introduced into the home cages of experimental mice for 4 min sessions. The latency to the first attack, the number of attacks, and the total duration of attacks initiated by the resident mice were recorded.

### 4.7. Tail-Flick Test

After acclimatization to the testing environment, mice were gently restrained within a holder with their tails exposed on a platform. A halogen lamp was positioned above the tail at minimal intensity sufficient to induce mild discomfort without causing tissue damage. The latency to tail flick, a rapid withdrawal response indicating discomfort, was automatically recorded by the Tail Flick Analgesia Meter system (Panlab, Barcelona, Spain), which simultaneously ceased the stimulus upon detection of movement.

### 4.8. Killing of Mice and Sample Collection

Mice were terminally anesthetized using a combination of CO_2_ and isoflurane, in accordance with previously established protocols [71,105,106]. Transcardial perfusion was then performed using 10 mL of ice-cold 0.9% NaCl. Following perfusion, brains were extracted, and the prefrontal cortex, hippocampus, amygdala, striatum, and dorsal raphe were dissected, rapidly frozen on dry ice, and stored at −80 °C until further use.

### 4.9. Quantitative Real-Time PCR

Total mRNA was extracted from each brain region using the RNeasy Lipid Tissue Mini Kit (Qiagen, Hilden, Germany). First-strand cDNA synthesis was performed using 1 μg of total RNA and the QuantiTect Reverse Transcription Kit (Qiagen, Hilden, Germany). qRT-PCR was conducted using SYBR Green Master Mix (Bio-Rad Laboratories, Philadelphia, PA, USA) on a ProFlex PCR system (Thermo Fisher Scientific, Waltham, MA, USA). Each 10 μL reaction contained 5 μL SYBR Green master mix, 3 μL RNase-free water, 1 μL of specific forward and reverse primers (20 pmol/μL), and 1 μL cDNA. Glyceraldehyde-3-phosphate dehydrogenase (Gapdh) was used as a reference gene due to its relatively stable expression in the brain [44,107]. The qRT-PCR protocol consisted of an initial denaturation at 95 °C for 4 min, followed by 40 cycles of denaturation at 95 °C for 20 s and annealing at 54 °C for 90 s. Primer sequences for genes of interest (Il-1β, Tnf, c-Fos, Cox-1, Cox-2) are provided in Supplementary Table S1. All samples were analyzed in triplicate. Gene expression was normalized to Gapdh and calculated as relative fold changes using previously established methods [44,102,104].

### 4.10. Protein Carbonyl Assay

The quantification of protein carbonyls was conducted utilizing the OxiSelect Protein Carbonyl Fluorometric Assay Kit (Cell Biolabs, Inc., San Diego, CA, USA). The samples were homogenized with a glass-glass homogenizer, followed by sonication on ice in 1 mL of 1× Sample Diluent provided by the kit. The homogenates were then centrifuged at 10,000 g for 5 min at 4 °C, and the supernatants were collected. Protein concentrations were adjusted to a range of 1–10 mg/mL using the Sample Diluent. Subsequently, protein carbonyl levels were quantified in accordance with the manufacturer’s instructions using a GloMax Multi Detection System (Promega, Madison, WI, USA) equipped with a fluorescence module (485/540 nm filter set). The results were normalized to protein concentration as previously described [15,44].

### 4.11. Total Glutathione Assay

The quantification of total glutathione content was conducted as previously described [44], utilizing the BioVision Glutathione Fluorometric Assay Kit (BioVision, Inc., San Francisco, CA, USA) in accordance with the manufacturer’s instructions. Each sample comprised approximately 40 mg of brain tissue, which was homogenized using a glass-glass homogenizer, followed by sonication on ice in 100 μL of ice-cold Glutathione Assay Buffer. Subsequently, total glutathione levels were assessed using the GloMax Multi Detection System (Promega, Madison, WI, USA) equipped with a fluorescence module (340/420 nm filter set).

### 4.12. Drug Administration

In this study, experimental solutions were utilized in place of standard drinking water. Thiamine or benfotiamine (Sigma-Aldrich, St. Louis, MO, USA) was dissolved in tap water at a concentration of 200 mg/kg/day, with the solutions being refreshed every 4–5 days, as previously documented [15,43,44]. The pH of the solutions was adjusted to 7.0. This method of administration and dosage was chosen based on prior research, which demonstrated no significant changes in total weekly fluid intake, as reported in other studies [15,44].

### 4.13. Statistical Analysis

Data analysis was executed utilizing GraphPad Prism software, version 8.01 for Windows (GraphPad Software, San Diego, CA, USA). Potential confounding variables were systematically controlled. No data points were excluded from the analysis. The Shapiro–Wilk test was employed to evaluate the normality of all quantitative datasets. For data conforming to a normal distribution, a two-way analysis of variance (ANOVA) was conducted, followed by Tukey’s post hoc test for multiple comparisons. In instances where data did not satisfy the normality assumption, the Kruskal–Wallis test was applied. One data point was removed from the tail flick test analysis as it was identified as an outlier using the Robust Regression and Outlier Removal (ROUT) method. Statistical significance was determined at *p* < 0.05. All results are reported as mean ± SD. Group sizes are specified in the figure legends.

## 5. Conclusions

Taken together, the mouse paradigm of rat exposure may be considered an etiologically relevant model of PTSD-like pathology with sound face validity and pharmacological sensitivity that recapitulates the key behavioral and molecular elements of this disease [62]. This model involves a sustained, inescapable threat from a natural predator, closely mimicking the chronic, life-threatening stressors experienced in humans; like other valid PTSD rodent paradigms, it mimics elevated anxiety, hypervigilance, neuroimmune alterations, and global increase in oxidative stress in the brain [62]. Similarly, treatment with thiamine or benfotiamine ameliorated most of these changes in the stressed groups. Thus, given the well-demonstrated role of oxidative stress in PTSD-like syndromes, these data can be interpreted as evidence of the pharmacological sensitivity of the model. Finally, the present findings, along with clinical evidence of excellent tolerability of thiamine and benfotiamine, suggest their beneficial therapeutic effects in patients with PTSD.

## Data Availability

Data are available upon request.

## Supplementary Materials

The supporting information can be downloaded at: https://www.mdpi.com/article/10.3390/ijms26146627/s1.
