# Supplementary material for: Thiamine Compounds Alleviate Oxidative Stress, Over-Expression of Pro-Inflammatory Markers and Behavioral Abnormalities in a Mouse Predation Model of PTSD
Source: Int J Mol Sci. 2025 Jul 10;26(14):6627. doi: 10.3390/ijms26146627 (PMC12294334; doi:10.3390/ijms26146627)
Supplement: Supplementary file 1 [file ijms-26-06627-s001.zip › ijms-3746092-supplementary.pdf]

Supplementary file

*Effects of thiamine and benfotiamine on aggression parameters in rat exposure model*

In the resident-intruder test, no significant group differences were found in the latency to attack, total number of attacks, and total duration of attacks (p=0.2424, p=0.2093, and p=0.308, respectively, Krushkal-Wallis test; Suppl. Fig.1A-C).

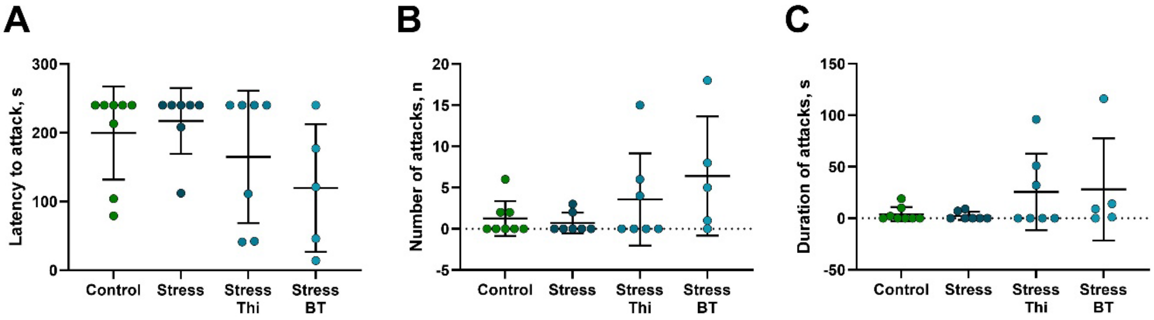


**Supplementary Figure 1.** No significant differences were revealed in **(A)** latency to attack, **(B)** total number of attacks, and **(C)** total duration of attacks. One-way ANOVA; 5–8 animals per group were used. Bars are Mean ± SEM. Thi – thiamine, BFT – benfotiamine.

**Supplementary Table S1.** Sequences of the primers for *Gapdh, Il-1β*, *Tnf, c-Fos, Cox-1,* and *Cox-2.*

| Gene | Forward sequence 5′-3′ | Reverse sequence 5′-3′ |
|---|---|---|
| *Gapdh* | TGCACCACCAACTGCTTAG | GGATGCAGGGATGATGTTC |
| *Il-1β* | CCTCCAGGATGAGGACATGAGCAC | TCATCATCCCATGAGTCACAGAGG |
| *Tnf* | AGCCGATGGGTTGTACCTTG | GTGGGGTGAGGAGCACGTAGTC |
| *c-Fos* | ATCCTTGGAGCCAGTCAAGA | ATGATGCCGGAAACAAGAAG |
| *Cox-1* | TCGGAGCCCCAGATATAGCA | TTTCCGGCTAGAGGTGGGTA |
| *Cox-2* | CCGTGCTGCTCTGTCTTAAC | TTGGGAACCCTTCTTTGTTC |
